# Tracing the origin of paracetamol tablets by near-infrared, mid-infrared, and nuclear magnetic resonance spectroscopy using principal component analysis and linear discriminant analysis

**DOI:** 10.1007/s00216-021-03249-z

**Published:** 2021-03-17

**Authors:** Alexander Becht, Curd Schollmayer, Yulia Monakhova, Ulrike Holzgrabe

**Affiliations:** 1 Institute for Pharmacy and Food Chemistry, University of Würzburg, Am Hubland, 97074 Würzburg, Germany; 2 Faculty of Chemistry and Biotechnology, Aachen University of Applied Sciences, 52428 Jülich, Germany; 3 Institute of Chemistry, Saratov State University, Astrakhanskaya Street 83, 410012 Saratov, Russia

**Keywords:** ^1^H NMR, IR, Manufacturer, Linear discriminant analysis, Principal component analysis

## Abstract

Most drugs are no longer produced in their own countries by the pharmaceutical companies, but by contract manufacturers or at manufacturing sites in countries that can produce more cheaply. This not only makes it difficult to trace them back but also leaves room for criminal organizations to fake them unnoticed. For these reasons, it is becoming increasingly difficult to determine the exact origin of drugs. The goal of this work was to investigate how exactly this is possible by using different spectroscopic methods like nuclear magnetic resonance and near- and mid-infrared spectroscopy in combination with multivariate data analysis. As an example, 56 out of 64 different paracetamol preparations, collected from 19 countries around the world, were chosen to investigate whether it is possible to determine the pharmaceutical company, manufacturing site, or country of origin. By means of suitable pre-processing of the spectra and the different information contained in each method, principal component analysis was able to evaluate manufacturing relationships between individual companies and to differentiate between production sites or formulations. Linear discriminant analysis showed different results depending on the spectral method and purpose. For all spectroscopic methods, it was found that the classification of the preparations to their manufacturer achieves better results than the classification to their pharmaceutical company. The best results were obtained with nuclear magnetic resonance and near-infrared data, with 94.6%/99.6% and 98.7/100% of the spectra of the preparations correctly assigned to their pharmaceutical company or manufacturer.

**Supplementary Information:**

The online version contains supplementary material available at 10.1007/s00216-021-03249-z.

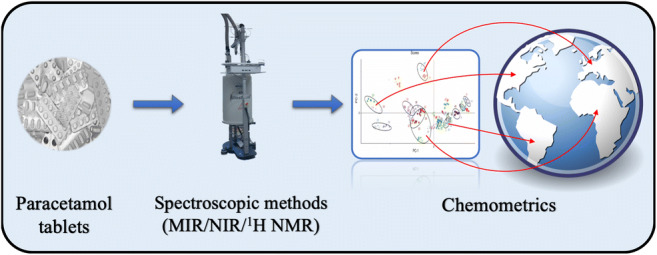

## Introduction

Nowadays, it is very difficult to determine the origin of a drug based on the declaration on primary or secondary packaging. One reason for this is the fact that a large number of counterfeit drugs are in circulation, not only in developing countries but also worldwide [1]. In addition to the actual medication, the packaging can be copied down to the last detail. Even the holograms can be counterfeited so perfect that these packages look more trustworthy than the original [2]. Another reason is that often many different companies are involved in the manufacturing process and most of them are not named in publicly available documents. In most cases, rather than the specific manufacturer, the marketing authorization holder is mentioned on the primary and secondary packaging, respectively. Research to figure out the manufacturer often leads to debatable websites. Even in developed countries, the manufacturer is not always clearly defined. For instance, in Germany, the pharmaceutical companies have to name the manufacturer in the package insert. However, these manufacturers just have to execute the last step in the production chain, which is the certification of the final product release. The excipients, the active pharmaceutical ingredient, or even the finished product can come from different manufacturing plants and suppliers from all over the world without the need of declaration. This makes it extremely difficult to determine the origin of a drug, although the identification of the manufacturer has a safety aspect for the patient, and also a commercial aspect for the pharmaceutical companies in terms of counterfeiting.

Chemometric methods are a common way to analyze large, complex spectral data and have found their way into various fields [3–12]. The combination of chemometric methods with near-infrared (NIR), mid-infrared (MIR), nuclear magnetic resonance (NMR), and Raman spectroscopy has solved many analytical challenges. It can be used to differentiate between organically and conventionally grown tomatoes in food chemistry, to evaluate complex samples in metabolomics studies, or to date documents in the forensic science [3–7], to name only a few applications. In the pharmaceutical sector, chemometric methods have also gained importance, for example, as a tool in process analytical technology [8, 9], counterfeit detection, and characterization of drug products or herbal medicines [10–12]*.*

The aim of this study was to identify the origin of drugs and the country of their manufacturing plant, respectively, solely based on measured spectra and the use of principal component analysis (PCA) and linear discriminant analysis (LDA). Since paracetamol formulations are widespread and easy to acquire, they have been used as a model product. Sixty-four paracetamol drug samples were purchased from 52 pharmaceutical companies from all parts of the world. Of these, 56 preparations formulated as tablets were used to generate three data sets using NIR, MIR, and NMR spectroscopy. With the help of PCA, an unsupervised method, the data was screened for patterns, which allows a tracing of the tablets back to their origin. In a second step and with the information gathered from PCA, the spectroscopic data were examined by LDA. Each of the spectral data sets was analyzed individually.

## Materials and methods

### Materials

Dimethylsulfoxide (D_6_, 99.8%) containing 0.03% (v/v) tetramethylsilane (TMS) and the 507-HP-7 5-mm routine NMR tubes were purchased from Euriso-top (Saarbrücken, Germany).

### Drug samples

All samples (see Table 1) were purchased in local pharmacies or in hospitals. Depending on the country, they were dispensed either in their original packaging or as single blister packs. Each tablet contained 500 mg of paracetamol and had different sizes, colors, or shapes. According to the labelling of some preparations, the types of excipients differed only slightly. The number of tablets contained in the marketed products varied between 4 and 30.

**Table 1:** Listing of all investigated paracetamol tablets. Samples marked with asterisk were used for the LDA models to predict the manufacturer and land of production. Sample nos. 2, 3, 45, 49, 54, 62, and 63 were deleted from the sample collection because of the large differences in the formulation (see “Spectral experiments”). *AUS*, Australia; DEU, Germany; *ITA*, Italy; *AUT*, Austria; *THA*, Thailand; *IDN*, Indonesia; *BGD*, Bangladesh; *TZA*, Tanzania; *CZE*, Czech Republic; *POL*, Poland; *GBR*, Great Britain; *ESP*, Spain; *RUS*, Russia; *MNG*, Mongolia; *CHN*, China; *HKG*, Hong Kong; *COL*, Colombia; *USA*, United States of America; *PRT*, Portugal

Sample No.	Name	Pharmaceutical Company	Origin	Quantity
1*	Panadol	GlaxoSmithKline plc.	AUS	12
4*	Paracetamol Tablets	Chemists’ Own	AUS	24
5*	Paracetamol	AFT Pharmaceuticals Ltd.	AUS	20
6*	Paracetamol	Priceline	AUS	20
7*	Paracetamol 500	1A Pharma GmbH	DEU	20
8*	Paracetamol ratiopharm	Ratiopharm GmbH	DEU	20
9*	Tachiprina	Angelini ACRAF SpA	ITA	30
10*	Paracetamolo Farmakopea	Farmakopea SpA	ITA	20
11*	Acetamol Adulti	Abiogen Pharma SpA	ITA	20
12*	Paracetamolo Sella	Laboratorio Chimico Farmaceutico “A. SELLA” S.r.l.	ITA	30
13*	Mexalen	Ratiopharm GmbH	AUT	10
14*	Paracetamol Genericon	Genericon Pharma GmbH	AUT	10
16	McXY Para	Millimed Co., Ltd.	THA	10
17*	Paracetamol 500	Kamol	THA	10
18*	Sanmol	P.t. Sanbe Farma	IDN	4
19	Paracetamol	P.t. Bernofarm	IDN	10
20	Paracetmaol	P.t. Phyto Kemo Agung Farma	IDN	10
21	Pamol	P.t. Interbat	IDN	4
22*	Panadol	GlaxoSmithKline plc.	IDN	10
23*	Dumin	P.t. Actavis Indonesia	IDN	10
24	Ace	Square Pharmaceuticals Ltd.	BGD	10
25	Napa	Beximco Pharmaceutials Ltd.	BGD	10
26	Paracetamol	Crescent Pharma Ltd.	BGD	10
27*	Vetocin	Nestor Pharmaceuticals Ltd.	TZA	10
28	Paracetamol	North China Pharmaceutical Co., Ltd.	TZA	10
29	Cetamol	Regal Pharmaceuticals Ltd.	TZA	10
30	Panadol Advance	GlaxoSmithKline plc.	TZA	10
31*	Asmol	Astra Lifecare (India) Pvt. Ltd.	TZA	10
32*	Dolomol	Lincoln Pharmaceuticals Ltd.	TZA	10
33*	Para-Denk 500	DENK PHARMA GmbH & Co. KG	TZA	10
34	Elymol	Elys Chemical Industries Ltd.	TZA	10
35*	Agomol	Agog Pharma Ltd.	TZA	10
36	Diodol	Keko Pharmaceutical Industries Ltd.	TZA	10
37*	Parakant	S Kant Healthcare Ltd.	TZA	10
38*	Paracetamol Dr.Max	Dr. Max Pharma Ltd.	CZE	30
39*	Paralen	Zentiva Group, a.s.	CZE	24
40*	Paracetamol Actavis	Actavis	POL	24
41	Paracetamol Polfa Lodz	Bio-Profil Polska Sp.z o.o / Laboratoria Polfa Łódź Sp. z o.o.	POL	10
42	Paracetamol	BIOFARM Sp. z o.o.	POL	20
43*	Paracetamol	STADA Arzneimittel AG	DEU	20
44*	Paracetamol AL 500	Aliud Pharma GmbH	DEU	20
46*	Paracetamol 500 mg elac	Inter Pharm Arzneimittel GmbH	DEU	20
47*	ben-u-ron	bene-Arzneimittel GmbH	DEU	20
48*	Paracetamol Hexal	Hexal AG	DEU	20
50*	Paracetamol	Aspar pharmaceuticals Ltd.	GBR	16
51*	Panadol Advance	GlaxoSmithKline plc.	GBR	16
52*	Paracetamol Winthrop	sanofi-aventis, S.A.	ESP	20
53*	Antidol	Laboratorios Cinfa S.A.	ESP	20
55*	Paracetamol	Renewal JSC	RUS	20
56*	Paracetamol	pharmstandard JSC	RUS	20
57*	Paracetamol	Nakhia Impex LLC	MNG	20
58	Panadol	tskf Co., Ltd.	CHN	10
59*	Panadol ActiFast	GlaxoSmithKline plc.	HKG	16
60	Acetaminofen	Laproff S.A.	COL	10
61	Tylenol	Johnson&Johnson Services, Inc.	USA	10
64*	Paracetamol Farmoz	Farmoz - Sociedade Técnico Medicinal, S.A.	PRT	20

### Sample preparation

For NIR and MIR measurements, the tablets were mortared and measured directly. For NMR experiments, an additional tablet per sample was mortared and placed in a falcon tube. Then, 6 mL of DMSO-*d*_6_ containing 0.03% TMS (v/v) as reference standard was added. The samples were vortexed (1 min), sonicated (1 h), and centrifuged (20 min/6k U/min). Six aliquots of the supernatant were analyzed by NMR spectroscopy (600 μL each).

Due to the small number of available tablets of some samples, therefore usually only the availability of one batch and the need for a whole tablet to obtain reproducible NMR spectra, only one tablet per company could be measured. For these reasons, an additional batch of the German preparations was acquired, measured, and compared with the first batch. It could be seen that the difference between the batches is very small for most companies, and therefore, the choice of sample preparation is acceptable, even if the validation is thus only valid for repeat measurements.

### Spectral experiments and analysis

The acquisition parameters for NMR, NIR, and MIR spectroscopy measurements were already reported in Belugina R. B. et al. [13].

#### NMR spectroscopy

All samples were analyzed with a Bruker Avance III 400 MHz spectrometer operating at 400.13 MHz with an inverse probehead. The ^1^H NMR experiments were measured at 300.11 ± 0.03 K with a 90° flip angle, 64 scans, no rotation, and an acquisition time of 5.45 s followed by a relaxation delay of 12 s. The receiver gain was set to 14.04 and a line broadening factor of 0.3 Hz was applied. The resulting digital resolution was 0.183 Hz over a spectral width of 30.04 ppm (time domain size 128k). Phasing and baseline correction were performed manually with TopSpin versions 3.5 and 4.0 (Bruker BioSpin GmbH, Rheinstetten, Germany). All signals were referred to the TMS signal. Each sample was measured six times.

#### NIR spectroscopy

Reflectance spectra were performed on a MicroNIR™ 1700 ES spectrometer with a windowed collar (VIAVI Solutions Deutschland GmbH, Eningen unter Achalm, Germany) covering a spectral range of 950–1650 nm. It works with two tungsten lamps and detection was performed with a photodiode array detector. The drug samples were measured by placing the glass vials on the windowed collar and rotating them after every measurement. Twelve spectra were recorded per sample with an average of 12 scans and an integration time of 12.2 s.

#### MIR spectroscopy

An FT/IR-6100 spectrometer (JASCO Deutschland GmbH, Pfungstadt, Germany) equipped with an attenuated total reflectance unit was used to acquire the MIR spectra. Twelve spectra of every drug sample were measured in a spectral range of 4000–550 cm^−1^ with 256 scans per spectrum and a resolution of 4 cm^−1^.

### Pre-processing and multivariate data analysis

For multivariate analysis, NMR spectra were reduced by bundling spectral regions of equal width of 0.04 ppm using Amix 3.9.15 (Bruker BioSpin GmbH, Rheinstetten, Germany). The spectral range from 0 to 11 ppm was used for further examination with PCA. The spectral regions of the residual water signal from 3.42 to 3.50 ppm, the residual dimethylsulfoxide signal from 2.34 to 2.70 ppm, and the TMS signal from −0.06 to 0.06 ppm were excluded. The final range used for PCA was 6.42–3.54 ppm, 3.38–2.74 ppm, 2.30–2.18 ppm, and 1.78–0.50 ppm.

Before further processing, the MIR-transmission spectra were transformed into absorption spectra and a baseline correction was applied. To remove scatter effects or compensate for additive effects from MIR and NIR data, an extended multiplicative scatter correction (EMSC) and a standard normal variate transformation (SNV) were applied, respectively. The first derivative was performed for both spectral data sets.

The final spectral range of interest was limited to 1175.401 to 861.0605 cm^−1^ for MIR and 1100.125–1242.595 nm and 1347.899–1570.896 nm for NIR spectra. All pre-processing methods and the individual analysis of the three spectral methods with PCA and LDA were performed with the Unscrambler X 10.4 (CAMO Software AS., Oslo, Norway). The permutation tests and data fusion analysis were performed with MATLAB 2016a (The MathWorks, Natick, MA). For the LDA, the prior probabilities were assumed to be equal and it was performed with the PCA scores due to the high number of variables [14]. The optimal number of scores was evaluated individually by comparing the results and accuracy of several LDA models, where the scores were successively reduced. For the LDA models of the NIR and MIR data, six or seven components must be used to obtain sufficient accuracy. Depending on the goal to determine the manufacturer or the pharmaceutical company, four or three components were sufficient to create the model with the NMR data. For the examination with PCA and LDA, not all variables were used, but only those of the final ranges defined above. The prediction performance of the LDA models was tested with a custom cross-validation for repeated measurements.

## Results and discussion

### Sample information

For most of the drugs, the information like their manufacturer or composition was noted on the primary or secondary packaging or in the package insert. For the remaining drugs, the websites of the authorization holders or the relevant national authorities were screened for further information about the manufacturer and the samples itself. As far as possible, information was also collected on the legal and business relations between the companies.

### Spectral experiments

Since paracetamol was the active pharmaceutical ingredient in each drug sample, the formulations differ mainly with regard to excipients and their amount. Because mainly tablets were available and the other formulations differed too much from them, only tablets with the same paracetamol content were considered (56 out of 64 samples). The other samples were discarded from the sample collection. However, the mass fraction of the API in the tablets ranged between 74 and 95% (w/w), which resulted in the paracetamol signals being the dominant part in the spectra. Exceptions were Paracetamol Polfa Lodz® (62%) and Panadol Acti Fast® (38%). The focus of the first steps was to identify the spectral fingerprints of each sample in every method.

**Fig. 1:**
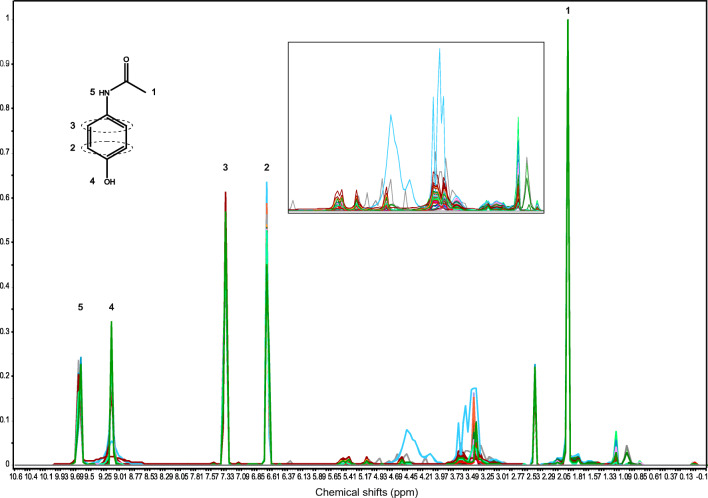
Reduced ^1^H NMR spectra of all paracetamol tablets with enlarged spectral range of the excipients (without residue signals of water, dimethylsulfoxide, and tetramethylsilane). The corresponding paracetamol signals are additionally marked. The spectra are color-coded according to the pharmaceutical companies

For multivariate data analysis, it is mandatory to have a sample preparation that generates reproducible spectra. Due to the variety of excipients in the tablets, e.g., large organic molecules alongside small inorganic molecules, it was difficult to find an appropriate solvent for NMR spectroscopy. DMSO was chosen because it was able to dissolve most of the excipients. Nevertheless, a residue often remained, which was centrifuged off. The sample preparation was identical for every tablet. Due to reproducibility issues caused by the one-sided ratio between paracetamol and excipients, it was necessary to measure the whole tablet and not just an aliquot. With the help of reference spectra, it was possible to identify the signals of paracetamol and of the excipients. The tablets mainly contained on average small amounts of cellulose derivatives, a type of starch (mostly maize starch), silica, stearic acid, povidone, and talcum. The spectral range in which the signals of the excipients appear was similar for all drug samples (0.5–6 ppm) and was therefore used for further PCA and LDA (reduced NMR spectra of tablets, see Fig. 1).

**Fig. 2:**
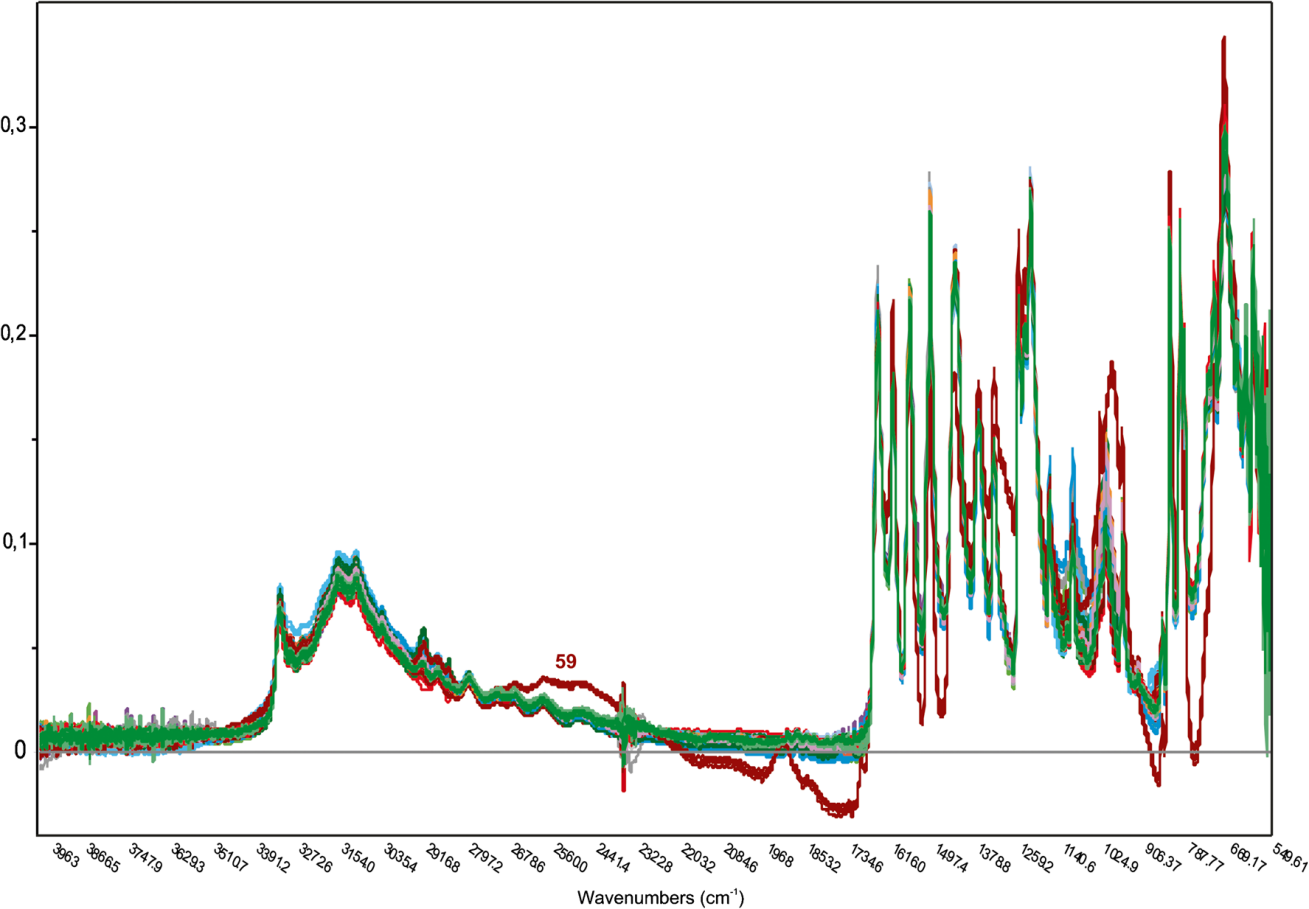
MIR spectra after transformation into absorbance spectra, baseline, and EMSC correction. Grouped by color according to the pharmaceutical companies. The marked spectrum is Panadol ActiFast (sample no. 59), which differs from the other spectra due to the additional high excipient content of sodium hydroxycarbonate

**Fig. 3:**
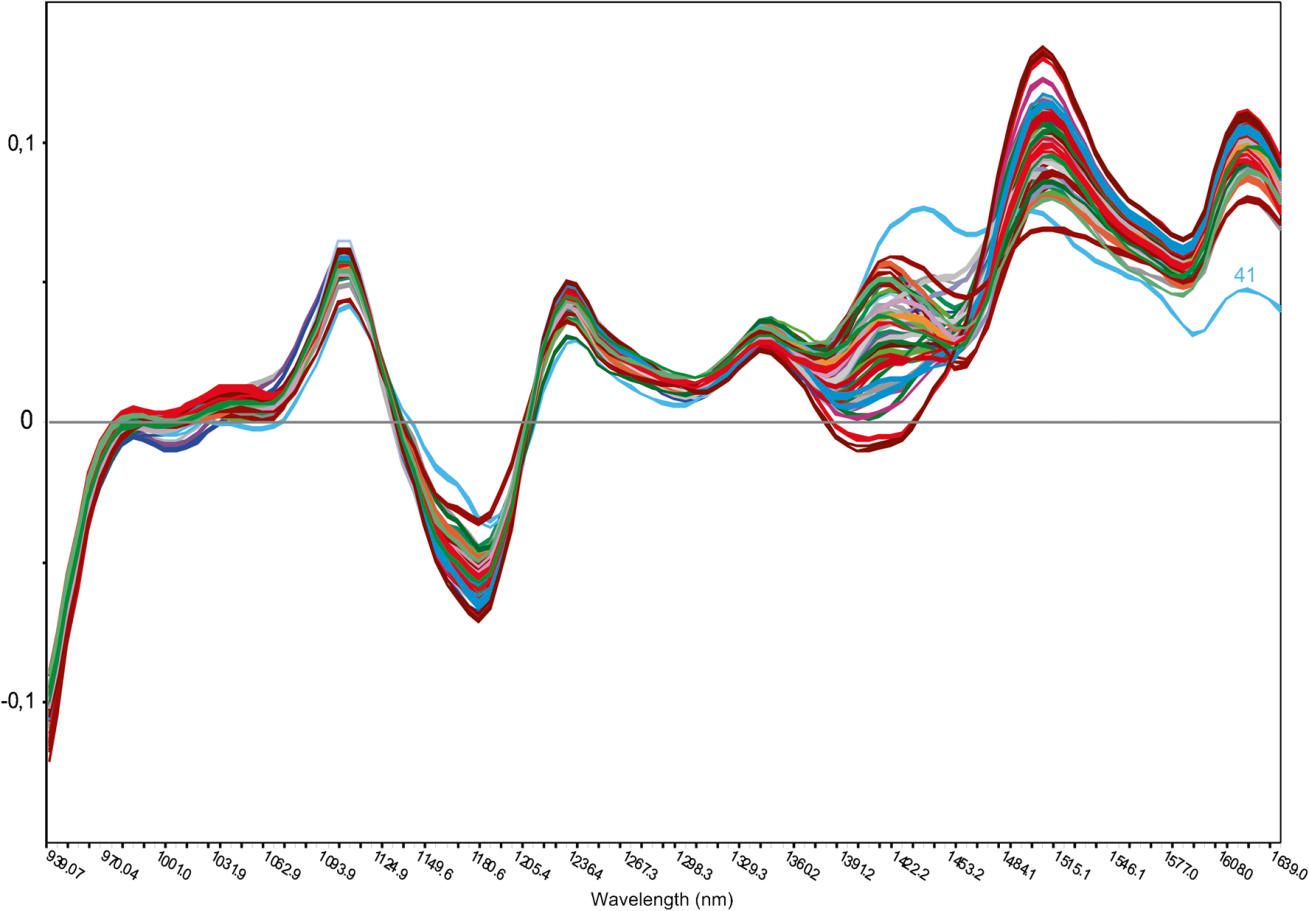
SNV-corrected NIR spectra (1st derivative). Grouped by color according to the pharmaceutical companies. The marked spectrum is Paracetamol Polfa Lodz (sample no. 41), which differs from the other spectra due to the additional high excipient content of sorbitol

MIR and NIR spectra (see Figs. 2 and 3) of the samples were very similar due to the high percentage of paracetamol. As expected, the only exceptions were Paracetamol Polfa Lodz (62% w/w) and Panadol ActiFast (38% w/w). This can be explained with their different formulations: they contain an additional amount of 170 mg sorbitol and 630 mg sodium hydroxycarbonate, respectively, which leads to the significant different spectra.

Because a specific device for the NIR instrument to directly measure the tablets was not available, the tablets had to be mortared. Physical information such as particle size or compression force can disappear as a result of this preparation step. Similar limitations hold true for the MIR spectra. However, after suitable pre-processing, a spectral range was found for both methods in which the spectra of the tablets differ.

### Principal component analysis

The main intention of multivariate data analysis is the extraction of useful information from the experimental data and revealing hidden relations as well as the reduction of the dimensionality of the spectral data to so-called principal components or latent variables [15]*.* One commonly used multivariate analysis technique is the principal component analysis (PCA). It is a projection method, which narrows the data dimensionality of several hundred or even thousand spectral values down to a few principal components and grants a better visualization of the data with appropriate plots. This allows for a better identification of the crucial spectral range, outliers, and clusters [16]. By means of the scores plot, in which the principal components are plotted against each other, the data sets were examined for clusters.

In a first review of the scores plots, two drug products were very noticeable. Depending on the spectroscopic method used, at least one of them was always clearly different from the other samples. These samples were Paracetamol Polfa Lodz and Panadol ActiFast. This was due to the high proportion of additional excipients, as already mentioned. For this reason, these samples had to be removed from the MIR data set to allow for a better evaluation of the other tablets, as they distorted the PCA too much.

**Fig. 4:**
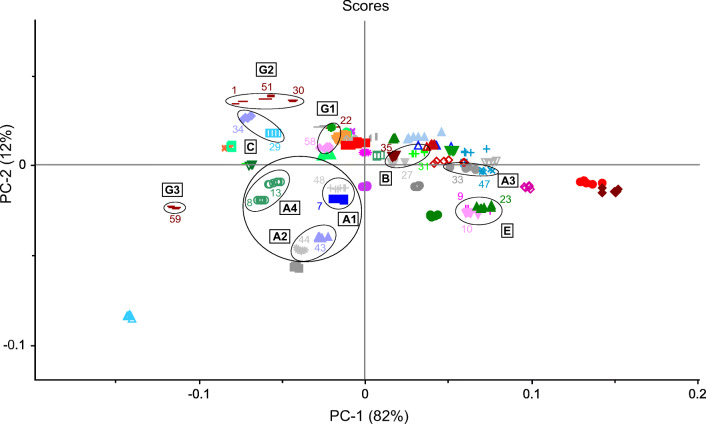
PCA of NIR spectra: 2D scatterplot with different colored symbols for each pharmaceutical company with the corresponding clusters as mentioned in “Principal component analysis.” The colored numbers correspond to the sample numbers from Table 1

**Fig. 5:**
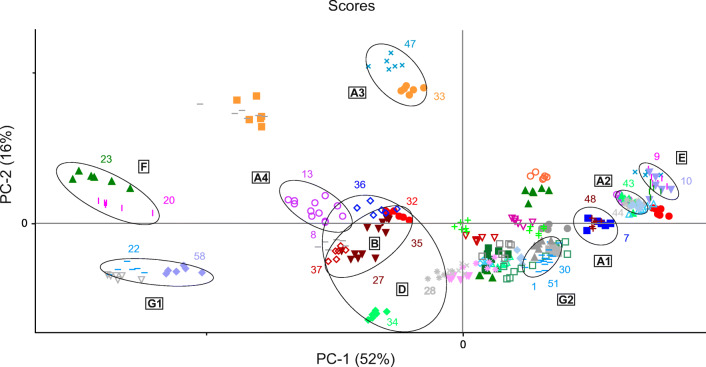
PCA of MIR spectra: 2D scatterplot with different colored symbols for each pharmaceutical company with the corresponding clusters as mentioned in “Principal component analysis.” The colored numbers correspond to the sample numbers from Table 1

**Fig. 6:**
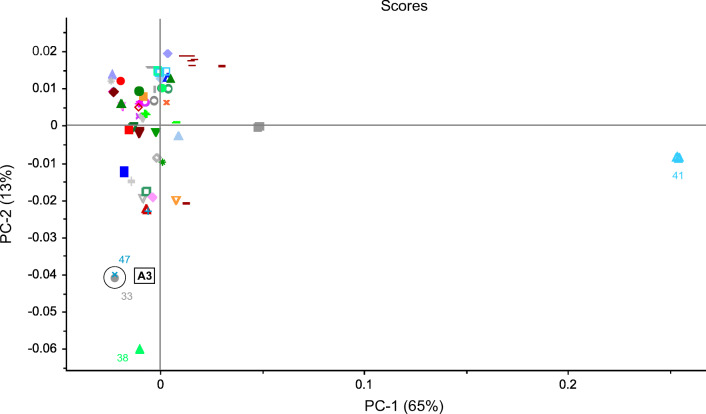
PCA of ^1^H NMR spectra: 2D scatterplot with different colored symbols for each pharmaceutical company with the corresponding clusters as mentioned in “Principal component analysis*.*” The colored numbers correspond to the sample numbers from Table 1

**Fig. 7:**
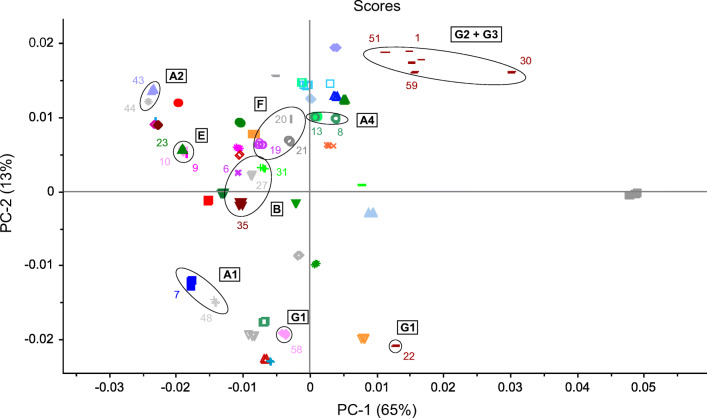
PCA of ^1^H NMR spectra: detail of the 2D scatterplot of Fig. 6 with the corresponding clusters as mentioned in “Principal component analysis.” Each colored symbol represents a pharmaceutical company and the colored numbers indicate the corresponding sample number (see Table 1)

For the remaining samples, cluster formation was observed for all three spectral methods (Figs. 4, 5, 6, and 7). The clusters were defined by the same manufacturer or country of origin and production, respectively. The analysis of the scores plots showed that some clusters were more differentiated from the other samples depending on the spectroscopic method. This was especially true for the MIR and NIR data. However, most of the clusters found in these plots were clearly separated from all other samples. Yet there was an accumulation of several samples, which could not be completely parted even in the higher PCs and therefore could not be assigned to specific clusters. Nevertheless, almost all of the determined clusters were found in all three scores plots, with the exception of two (clusters C and D, see below). These could only be seen using the NIR data (see Fig. 4). The identified clusters are listed below with the corresponding sample numbers in parentheses:

- Clusters of samples produced by the same manufacturer (Fig. 4, 5, 6, and 7): cluster A1: Hexal AG (48) and 1A Pharma GmbH (7)/cluster A2: STADA Arzneimittel AG (43) and Aliud Pharma GmbH (44)/cluster A3: bene-Arzneimittel GmbH (47) and Denk Pharma GmbH & Co. KG (33)/cluster A4: two drugs of Ratiopharm GmbH (from Germany (8) and Austria (13))
- Cluster B (bought in Tanzania but manufactured in India): Nestor Pharm. Lim. (27), Agog Pharma Ltd. (35), Lincoln Pharma (32), and S Kant Healthcare (37) (MIR; Fig. 5); Nestor Pharm. Lim., Agog Pharma Ltd., Astra Lifecare (India) Pvt. Ltd. (31) (NIR and NMR; Figs. 4 and 7). In addition, the NMR spectra cluster included a Priceline preparation (6), which originates from Australia, and also was manufactured in India.
- Cluster C (bought in Tanzania but manufactured in Kenya): Elys Chemical Industries Ltd. (34) and Regal Pharm. Ltd. (29) (NIR; Fig. 4)
- Cluster D (bought in Tanzania): Nestor Pharm. Lim., S Kant Healthcare, Agog Pharma Ltd., Lincoln Pharma, and Keko Pharm. Industries Ltd. (36). The samples from Elys Chemical Industries Ltd. and North China Pharm. (28) were a little further away, but still in the vicinity (MIR; Fig. 5)
- Cluster E (bought and manufactured in Italy): Angelini ACRAF SpA (9) and Farmakopea SpA (10) (MIR; Fig. 5). In the evaluation of the NIR and NMR spectra, PT. Actavis Indonesia (23) was additionally present (Figs. 4 and 7). Further investigations have shown that Farmakopea SpA belongs to the Unifarm group (subsidiary E-Pharma), based in Italy [17]. This group in turn produces pharmaceutical products for many companies, including Actavis and Angelini [18].
- Cluster F (bought and manufactured in Indonesia): PT. Actavis Indonesia and P.t. Phyto Kemo Agung Farma (20) (MIR; Fig. 5); PT. Interbat Pharmaceutical Industry (21), P.t. Phyto Kemo Agung Farma and Bernofarm (19) or PT. Interbat Pharmaceutical Industry and P.t. Sanbe Farma or PT. Actavis Indonesia and Bernofarm (NMR; depending on the PCs); PT. Interbat Pharmaceutical Industry and Bernofarm or PT. Interbat Pharmaceutical Industry and P.t. Sanbe Farma (PT. Actavis Indonesia and Bernofarm only in the vicinity) (NIR; higher PCs)
- Clusters of Panadol® preparations from GlaxoSmithKline (GSK): The sample set contained six different tablets of this brand, five from GSK and one from tskf. The latter is a joint venture between GSK and other pharmaceutical companies [19]. The preparations most likely came from four different manufacturers from Ireland (30, 51, 59), Australia (1), China (58), and Indonesia (22) and correspond to three different formulations: Panadol® (cluster G1; 22, 58), Panadol® Advance/Optizorb (cluster G2; 1, 30, 51), and Panadol® ActiFast (cluster G3; 59). These samples formed three clusters in the scores plot of the MIR and NIR spectra according to their formulation (Figs. 3 and 7). The PCA of the NMR spectra mainly differentiated between the standard formulation and those with a modified drug formulation (Fig. 7). Furthermore, it was also possible to distinguish between the manufacturing sites of the two standard preparations. A complete differentiation of all GSK samples according to their manufacturing site could be achieved by an individual PCA of the NIR and NMR spectra (for the corresponding scores plots, see Figs. S1–S4 in the Supplementary Information (ESM)). The information about the different tablet formulations was contained in the first principal components, whereas the information about the plant of manufacture was in the higher ones.

All in all, the three methods have produced valuable results, and in some cases, they have complemented each other. However, the main difference between them was the effort required for sample preparation. For the infrared spectra, the tablets had only to be mortared, whereas for the NMR spectra, first a suitable sample preparation and measurement method had to be established.

However, in the PCA of NMR spectra, it was possible to quickly and easily identify which excipients correlate significantly with the main components by means of the loadings (see Fig. S5 in the ESM for the loadings plots). From this, it can be seen which excipients are characteristic for the individual preparations. Due to the high proportion of sorbitol in Paracetamol Polfa Lodz® compared to the other excipients in the remaining preparations, the loadings of PC1 correspond to the signals of sorbitol. This also explains why PC1 mainly describes Polfa Lodz®. The second PC is determined by magnesium stearate, which appears to be present in large quantities at Dr. Max Pharma Ltd. (38), bene-Arzneimittel GmbH (47), and DENK PHARMA GmbH & Co. KG (33). PC3 correlates mainly with the starch derivatives, but to a certain extent it has additionally a positive correlation with lactose and a negative one with sorbitol and hydroxypropyl cellulose or hydroxypropyl(methyl)cellulose. PC4, on the other hand, has a strong positive correlation with the cellulose derivatives mentioned, which are contained in higher amounts in the preparations P.t. Sanbe Farma (18), PT. Interbat Pharmaceutical Industry (21), and Actavis (40). In the last PC, povidone has the highest weight, whereas this excipient is contained in almost all preparations and therefore contributes less to the differentiation of the drug samples.

### Linear discriminant analysis

In contrast to PCA, linear discriminant analysis (LDA) is a supervised method. The samples are first assigned to individual groups [20] before the parameters of the discriminant function are chosen in such a way that the differences within a group become minimal and maximal to the others [21]. The resulting model can then be used to make predictions about unknown samples and assign them to one of the previously defined groups.

First, different LDA models were created and compared to each other to test which categories are well predictable. Attempts were made to predict the pharmaceutical company, the manufacturer, or the country of production or origin, respectively. For the categories manufacturer and country of production, only those samples were used, for which this information could be verified by means of the blister or the package leaflet or via the website of the pharmaceutical company (s. Table 1). In addition, as in the PCA, the sample Panadol ActiFast was removed from the MIR data set for the determination of the pharmaceutical companies because its formulation is very different in comparison to the other tablets. This led to a much better classification for the remaining companies.

To ensure that the models are suitable for their intended purpose, a custom cross-validation was carried out to get an impression of the performance of the model. Therefore, the data sets were divided into six different test sets and model-building sets. For MIR and NIR data, eight spectra were used for the model-building set and two for the validation set. To get six different sets, the two spectra for validation testing were switched with two other spectra from the model-building set. The selection was limited to 10 spectra, since PCA could identify one or two outliers in some samples. This concerned the following sample numbers: NIR: 16, 31, 39, 52; MIR: 11, 38, 41, 55 (52, 55 with two outliers, the rest with one). For NMR data, five spectra were used to build the model and one for the prediction, always using a different spectrum for the prediction. The average percentage of correctly assigned spectra for every model is listed in Table 2.

**Table 2:** LDA results (average number of correctly assigned spectra in percentage after six LDAs) of different spectroscopic methods in relation to two different categories: manufacturers (35 different; 40 samples) and pharmaceutical companies (50 different; 56 samples). For MIR pharmaceutical companies, only 55 samples were used, as described in “Linear discriminant analysis”

	MIR	NIR	^1^H NMR
Manufacturers (*n* = 40)	91 (LDA1)	100 (LDA3)	99 (LDA5)
Pharmaceutical companies (*n* = 56)	89 (LDA2)	99 (LDA4)	99 (LDA6)

It was soon apparent that it is hardly possible to determine the country in which the preparations were acquired or in which they were produced. In some cases, far less than 60% correct assignments for MIR and NMR models were achieved. Only the NIR model was able to make between 60 and 70% correct classifications. This is not surprising, however, since the drugs are manufactured according to the companies’ specifications and not those of the countries. The fact that in PCA within some countries some preparations of different manufacturers are nevertheless very similar could be due to the local suppliers of excipients and the low variability in the compositions. For this reason, we have focused more on the other two categories being pharmaceutical company and manufacturer.

For the manufacturer and the pharmaceutical company, very good results were achieved (see Table 2), and therefore, an additional permutation test was performed [22]. This is a randomization test to check whether the chosen descriptors, like wavenumbers, are truly correlated to the response variable and does not lead to a correct selection just by chance [23]. For this purpose, the assignment of manufacturers or pharmaceutical companies to the spectra was scrambled and the percentage of the correct classification was compared to the original assignment. As can be seen in Table 3, the original correct classification rate is significantly higher for all three data sets than the one after scrambling.

**Table 3:** Results of permutation tests for three data sets regarding manufacturers and pharmaceutical companies. The percentage of correct classification is shown

	MIR	NIR	^1^H NMR
Manufacturers	59	69	74
Pharmaceutical companies	71	85	93

As can be seen in Table 2, the NMR and NIR methods were most capable of providing a correct classification. The LDA models based on the NIR data were able to correctly assign the spectra to both the manufacturer (LDA3) and the pharmaceutical companies (LDA4). Among the pharmaceutical companies, only Biofarm (42) had three wrong assignments for the 6 LDAs, as two wrong assignments for Hexal AG (48) and GlaxoSmithKline (1). The NMR models achieved very similarly results. Only the model for the determination of the pharmaceutical companies could not correctly determine three samples with any of the models. These were two samples from GlaxoSmithKline (22, 51) and one from Actavis (23). The MIR models (LDA1; LDA2) gave with around 90% correct classified spectra for both categories a little worse result for the classification. The biggest problems in the assignment of the pharmaceutical manufacturer (LDA2) were found in the spectra of GlaxoSmithKline (22), Farmoz (64), and Johnson&Johnson (61). Furthermore, the method was not able to distinguish between Aliud Pharma (44) and STADA (43), as well as Angelini (9) and Farmakopea (10). The latter also caused problems with the model for determining the manufacturer (LDA1), as did another sample from GlaxoSmithKline (51) and Farmoz (64).

A closer look revealed the reason of these incorrect assignments. The tablets of Aliud Pharma and STADA are both produced by STADA, which is why mix-ups occurred when determining the pharmaceutical company. Farmakopea and Angelini also seem to be connected via the Unifarm Group, as clarified in “Principal component analysis”—Cluster E. The problems of the GSK samples are due to the different formulations, which is why the models have problems assigning them to the same manufacturer. However, by further subdividing the pharmaceutical companies into manufacturers, the results can be improved and the models are better able to make classifications as seen for all spectral data sets. Overall LDA3 showed the best results. It was able to differentiate between all production sites of GSK, despite different formulations, and due to the clarification of the manufacturers, no more mix-ups occurred.

## Conclusion

It has been shown that chemometric evaluation of mid-infrared (MIR), near-infrared (NIR), and nuclear magnetic resonance (NMR) spectra using principal component analysis (PCA) and linear discriminant analysis (LDA) can be very useful in characterizing drugs and determining their origin. It was possible to identify relationships between companies and suppliers and to detect major differences or similarities in formulations. In addition, most of the samples could be assigned to their manufacturer or pharmaceutical company. Nevertheless, some points must be taken into account. This includes cooperations or mergers of companies, different production sites, or different formulations of the pharmaceutical companies. However, as is often the case with the manufacturer itself, this information is not listed or is difficult to retrieve and can lead to falsified correct or incorrect classifications.

The country of manufacture or country of origin could not be determined exactly by means of LDA, as this depends on too many factors, above all the fact that the drugs are of course not produced according to the specifications of the countries but of the respective companies. Added to this is the low variability in the formulations as well as the worldwide marketing of excipients that are rarely purchased locally or only by one company. However, the PCA shows that there may be some similarities between products from the same country. If an unknown sample is projected onto the PCA and it is inside the borders of a particular cluster (at a given probability), it can be assumed a new sample may also originate from that country.

When comparing the spectroscopic methods, NIR and NMR are preferred. With NIR, the sample preparation is very easy, the acquisition of the spectra is very fast, and the results are valid. With NMR, on the other hand, the sample preparation is more difficult but the classifications led to almost the same results, especially for the manufacturers. Furthermore, it is possible to obtain information about the composition of the different samples, allowing them to be characterized and then compared with other or unknown samples. However, it was shown that the information of all three methods can complement each other and that there is a benefit in using and analyzing with different spectral methods. Therefore, a further analysis using data fusion, where the spectral information of all methods is combined and analyzed simultaneously, would be beneficial. Our preliminary studies have shown that data fusion approach, namely, common components and specific weights analysis (CCSWA) [24, 25], can be used to differentiate paracetamol producer and marketing authorization holder (MAH). In this case, the percentage of correct classification varied between 93 and 96%. Similarly to the findings described in this study, NMR was proven to be the best method to detect paracetamol origin; the other data sets tend to worsen the model.

Since there is no complete disclosure of the pharmaceutical companies about the origin of the tablets or excipients, only assumptions can be made about some relations. If there were a better traceability, it should be possible to make even more precise statements about the origin with this method.

## Supplementary information

ESM 1 (PDF 456 kb)

ESM 2 (XLSX 52 kb)

## Data Availability

The data sets can be provided by request.

## Abbreviations

PCA: Principal component analysis
LDA: Linear discriminant analysis
NIR: Near-infrared
MIR: Mid-infrared
NMR: Nuclear magnetic resonance

## References

[1] Cockburn R, Newton PN, Agyarko EK, Akunyili D, White NJ (2005). The global threat of counterfeit drugs: why industry and governments must communicate the dangers. PLoS Med.

[2] Aldhous P (2005). Counterfeit pharmaceuticals: murder by medicine. Nature..

[3] Carolina S. Silva MFP, José Manuel Amigo, Carmen Garcia-Ruizc, Fernando Ortega-Ojedac. Infrared spectroscopy and chemometrics to evaluate paper variability in document dating. Spectrosc Eur 2018; 30(5):12–15.

[4] Gu H, Pan Z, Xi B, Hainline BE, Shanaiah N, Asiago V (2009). 1H NMR metabolomics study of age profiling in children. NMR Biomed.

[5] Hohmann M, Monakhova Y, Erich S, Christoph N, Wachter H, Holzgrabe U (2015). Differentiation of organically and conventionally grown tomatoes by chemometric analysis of combined data from proton nuclear magnetic resonance and mid-infrared spectroscopy and stable isotope analysis. J Agric Food Chem.

[6] Hohmann M, Christoph N, Wachter H, Holzgrabe U (2014). 1H NMR profiling as an approach to differentiate conventionally and organically grown tomatoes. J Agric Food Chem.

[7] Mao H, Wang H, Wang B, Liu X, Gao H, Xu M (2009). Systemic metabolic changes of traumatic critically ill patients revealed by an NMR-based metabonomic approach. J Proteome Res.

[8] Challa S, Potumarthi R (2013). Chemometrics-based process analytical technology (PAT) tools: applications and adaptation in pharmaceutical and biopharmaceutical industries. Appl Biochem Biotechnol.

[9] Matero S, van Den Berg F, Poutiainen S, Rantanen J, Pajander J (2013). Towards better process understanding: chemometrics and multivariate measurements in manufacturing of solid dosage forms. J Pharm Sci.

[10] Custers D, Cauwenbergh T, Bothy JL, Courselle P, De Beer JO, Apers S (2015). ATR-FTIR spectroscopy and chemometrics: an interesting tool to discriminate and characterize counterfeit medicines. J Pharm Biomed Anal.

[11] Li L, Zang H, Li J, Chen D, Li T, Wang F (2014). Identification of anisodamine tablets by Raman and near-infrared spectroscopy with chemometrics. Spectrochim Acta A Mol Biomol Spectrosc.

[12] Said MM, Gibbons S, Moffat AC, Zloh M (2011). Near-infrared spectroscopy (NIRS) and chemometric analysis of Malaysian and UK paracetamol tablets: a spectral database study. Int J Pharm.

[13] Belugina RB, Monakhova YB, Rubtsova E, Becht A, Schollmayer C, Holzgrabe U (2020). Distinguishing paracetamol formulations: comparison of potentiometric “Electronic Tongue” with established analytical techniques. J Pharm Biomed Anal.

[14] Bertrand D, Courcoux P, Autran J-C, Meritan R, Robert P (1990). Stepwise canonical discriminant analysis of continuous digitalized signals: application to chromatograms of wheat proteins. J Chemom.

[15] Schönberger T, Monakhova YB, Lachenmeier DW, Walch S, Kuballa T, et al. Guide to NMR method development and validation – part II: multivariate data analysis. Eurolab Technical Report No 01/2015. 2015.

[16] Biancolillo A, Marini F. Chemometric methods for spectroscopy-based pharmaceutical analysis. 2018; 6(576). 10.3389/fchem.2018.00576 PMC6258797 30519559

[17] Unifarm. Information about the Unifarm group. https://www.unifarm.it/it/gruppo/. 2020 Accessed 15 April 2020.

[18] E-Pharma. Business partners of E-Pharma. https://www.e-pharma.com/en/health/partners. 2020 Accessed 15 April 2020.

[19] GSK China. Information about GSK in China. https://www.gsk-china.com/en-gb/about-us/gsk-in-china/. 2020 Accessed 21 April 2020.

[20] Cozzolino D, Chree A, Scaife JR, Murray I (2005). Usefulness of near-infrared reflectance (NIR) spectroscopy and chemometrics to discriminate fishmeal batches made with different fish species. J Agric Food Chem.

[21] Martinez AM, Kak AC (2001). PCA versus LDA. IEEE Trans Pattern Anal Mach Intell.

[22] Lindgren F, Hansen B, Karcher W, Sjöström M, Eriksson L (1996). Model validation by permutation tests: applications to variable selection. J Chemom.

[23] Rücker C, Rücker G, Meringer M (2007). y-Randomization and its variants in QSPR/QSAR. J Chem Inf Model.

[24] Monakhova YB, Hohmann M, Christoph N, Wachter H, Rutledge DN (2016). Improved classification of fused data: synergetic effect of partial least squares discriminant analysis (PLS-DA) and common components and specific weights analysis (CCSWA) combination as applied to tomato profiles (NMR, IR and IRMS). Chemom Intell Lab Syst.

[25] Qannari EM, Wakeling I, Courcoux P, MacFie HJH (2000). Defining the underlying sensory dimensions. Food Qual Prefer.

