# Supplementary material for: Tracing the origin of paracetamol tablets by near-infrared, mid-infrared, and nuclear magnetic resonance spectroscopy using principal component analysis and linear discriminant analysis
Source: Anal Bioanal Chem. 2021 Mar 17;413(11):3107–18. doi: 10.1007/s00216-021-03249-z (PMC8043955; doi:10.1007/s00216-021-03249-z)
Supplement: Supplementary file 1 — (PDF 456 kb) [file 216_2021_3249_MOESM1_ESM.pdf]

Tracing the origin of paracetamol tablets by near-infrared, mid-infrared and nuclear magnetic resonance spectroscopy using principal component analysis and linear discriminant analysis

Alexander BECHT[a], Curd SCHOLLMAYER[a], Yulia MONAKHOVA[b,c], Ulrike HOLZGRABE[a,*]

[a] University of Würzburg, Institute for Pharmacy and Food Chemistry, 97074 Würzburg, Germany

[b] Aachen University of Applied Sciences, Faculty of Chemistry and Biotechnology, 52428 Jülich, Germany

[c] Institute of Chemistry, Saratov State University, Astrakhanskaya Street 83, 410012 Saratov, Russia

* corresponding author: Prof. Dr. Ulrike Holzgrabe
Institute for Pharmacy and Food Chemistry
University of Würzburg
Am Hubland
97074 Würzburg
Phone: +49 931 31 85460.
Fax: +49 931 31 85494
E-mail address: ulrike.holzgrabe@uni-wuerzburg.de

Supplementary Information

Contents:

Part 1

Fig. S1 Scores plot of all GlaxoSmithKline samples clustered according to the country of their manufacturing site based on the NIR data. The colored numbers correspond to the sample numbers from Tab. 1 and in color to the manufacturing site in the legend (AUS: Australia; CHN: China; IDN: Indonesia; IRL: Ireland).

Fig. S2 Scores plot of all GlaxoSmithKline samples clustered according to their formulation (Panadol®: 22, 58; Panadol® ActiFast: 59; Panadol® Advanced/Optizorb: 1, 30, 51) based on the NIR data. The colored numbers correspond to the sample numbers from Tab. 1 and in color to the manufacturing site in the legend (AUS: Australia; CHN: China; IDN: Indonesia; IRL: Ireland).

Fig. S3 Scores plot of all GlaxoSmithKline samples clustered according to the country of their manufacturing site based on the NMR data. The colored numbers correspond to the sample numbers from Tab. 1 and in color to the manufacturing site in the legend (AUS: Australia; CHN: China; IDN: Indonesia; IRL: Ireland).

Fig. S4 Scores plot of all GlaxoSmithKline samples clustered according to their formulation (Panadol®: 22, 58; Panadol® ActiFast: 59; Panadol® Advanced/Optizorb: 1, 30, 51) based on the NMR data. The colored numbers correspond to the sample numbers from Tab. 1 and in color to the manufacturing site in the legend (AUS: Australia; CHN: China; IDN: Indonesia; IRL: Ireland).

Fig. S5 Loadings Plots of the first four principal components of the PCA of NMR data with the help of which the excipients could be identified, which correlate mainly with the respective principal component

Part 2

Part 2 (see separate Excel file) contains a representative confusion matrix for each category and spectral method (six in total).

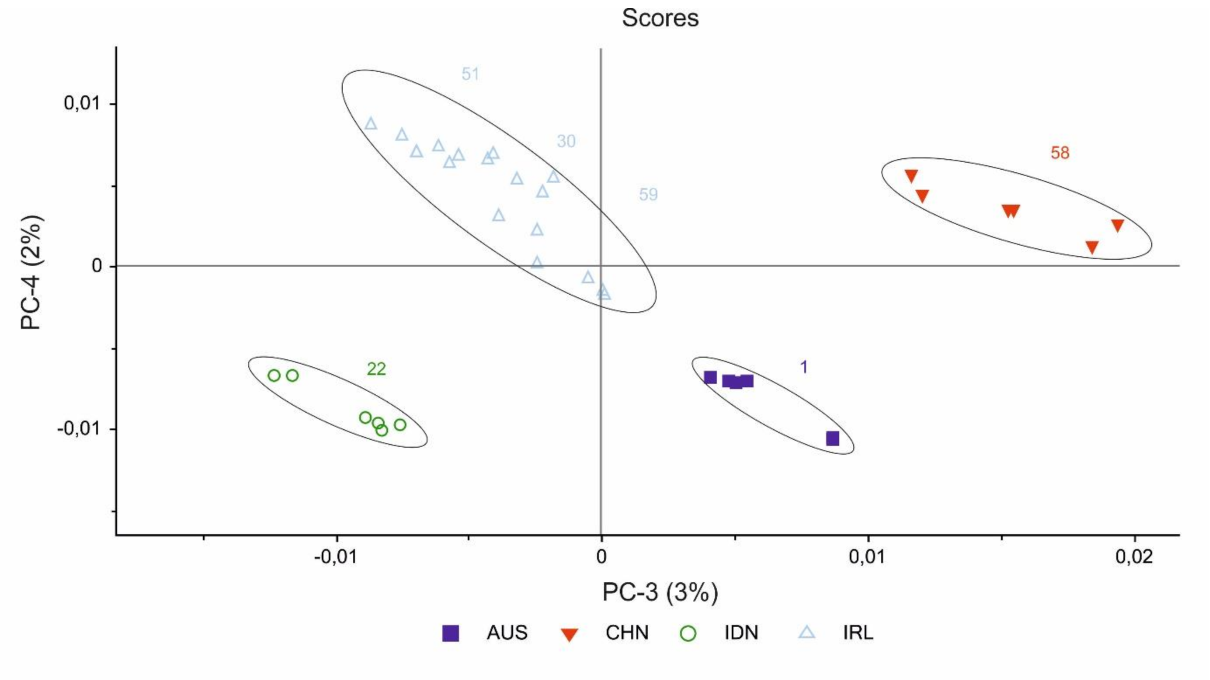

Fig. S1 Scores plot of all GlaxoSmithKline samples clustered according to the country of their manufacturing site based on the NIR data. The colored numbers correspond to the sample numbers from Tab. 1 and in color to the manufacturing site in the legend (AUS: Australia; CHN: China; IDN: Indonesia; IRL: Ireland).

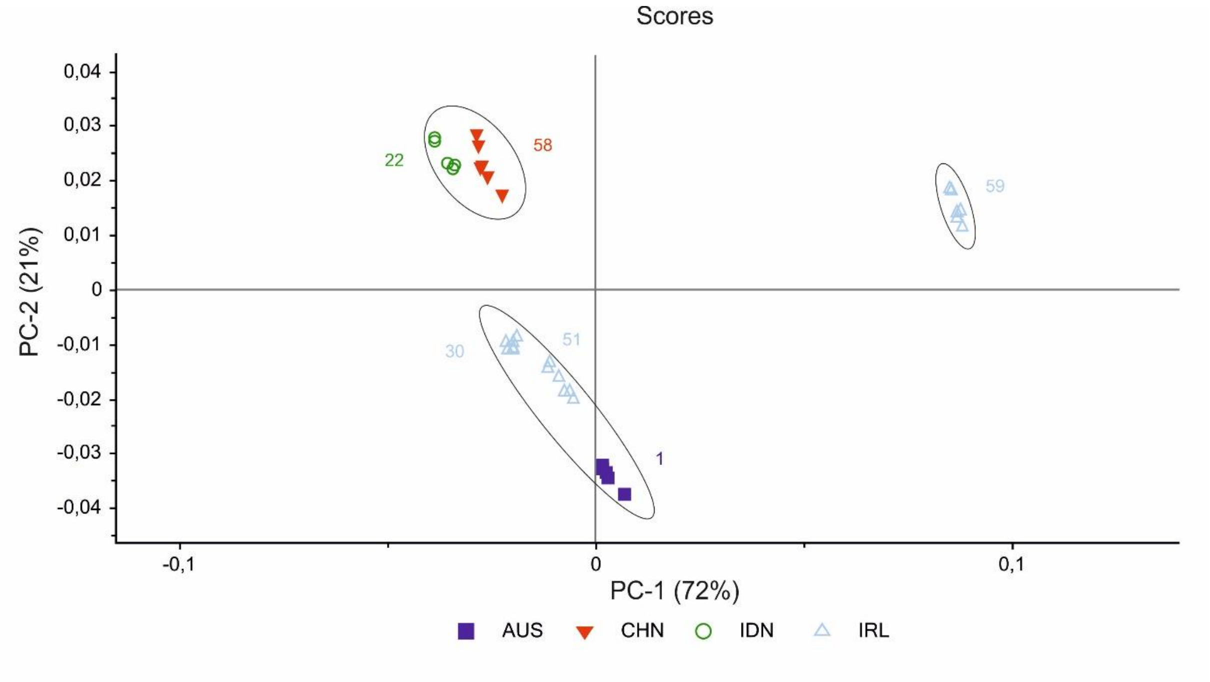

Fig. S2 Scores plot of all GlaxoSmithKline samples clustered according to their formulation (Panadol®: 22, 58; Panadol® ActiFast: 59; Panadol® Advanced/Optizorb: 1, 30, 51) based on the NIR data. The colored numbers correspond to the sample numbers from Tab. 1 and in color to the manufacturing site in the legend (AUS: Australia; CHN: China; IDN: Indonesia; IRL: Ireland).

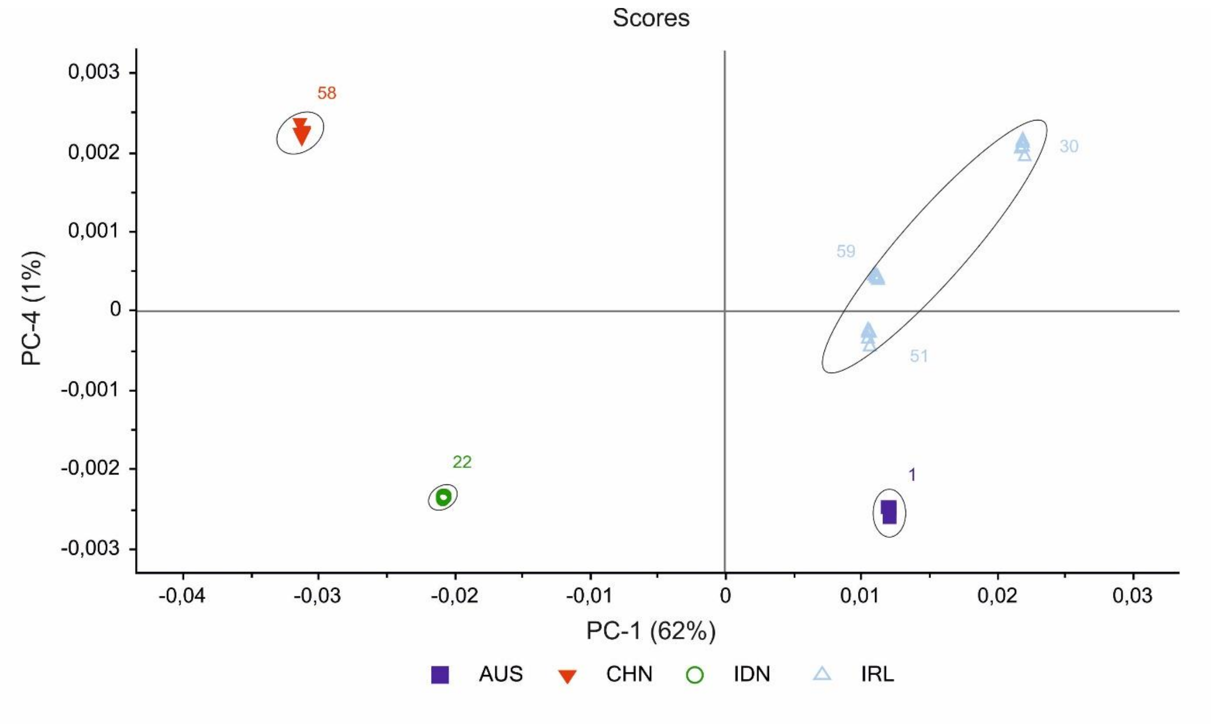

Fig. S3 Scores plot of all GlaxoSmithKline samples clustered according to the country of their manufacturing site based on the NMR data. The colored numbers correspond to the sample numbers from Tab. 1 and in color to the manufacturing site in the legend (AUS: Australia; CHN: China; IDN: Indonesia; IRL: Ireland).

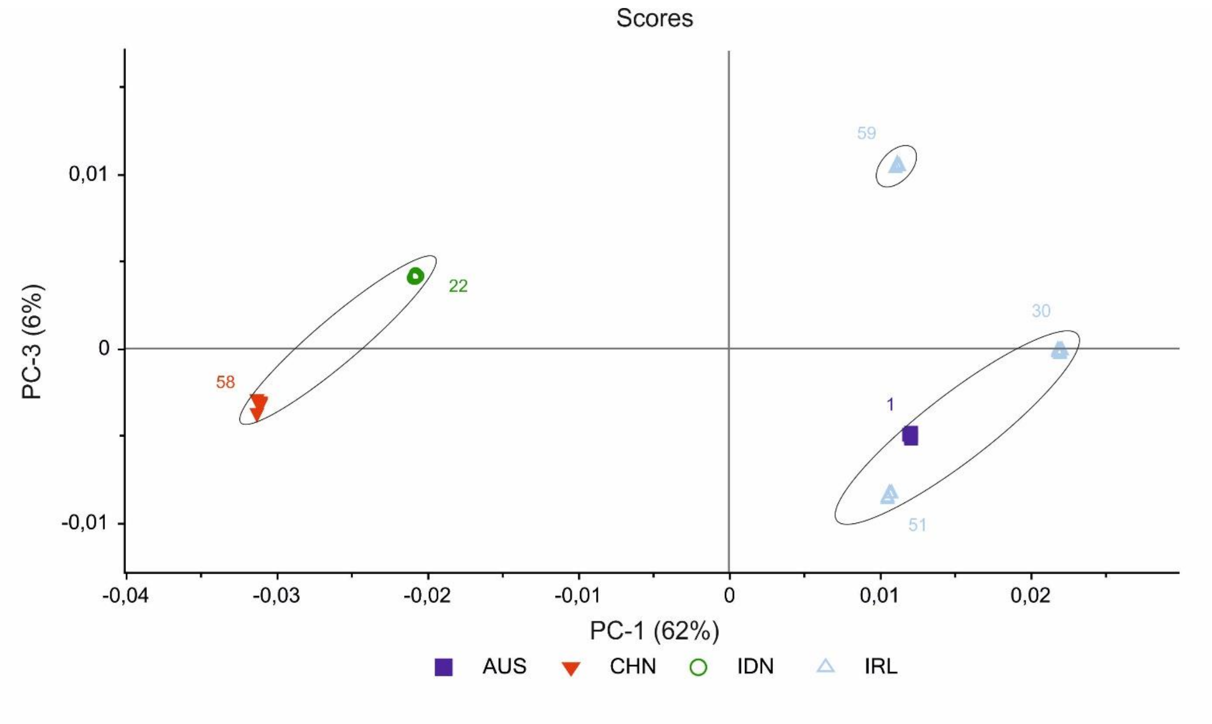

Fig. S4 Scores plot of all GlaxoSmithKline samples clustered according to their formulation (Panadol®: 22, 58; Panadol® ActiFast: 59; Panadol® Advanced/Optizorb: 1, 30, 51) based on the NMR data. The colored numbers correspond to the sample numbers from Tab. 1 and in color to the manufacturing site in the legend (AUS: Australia; CHN: China; IDN: Indonesia; IRL: Ireland).

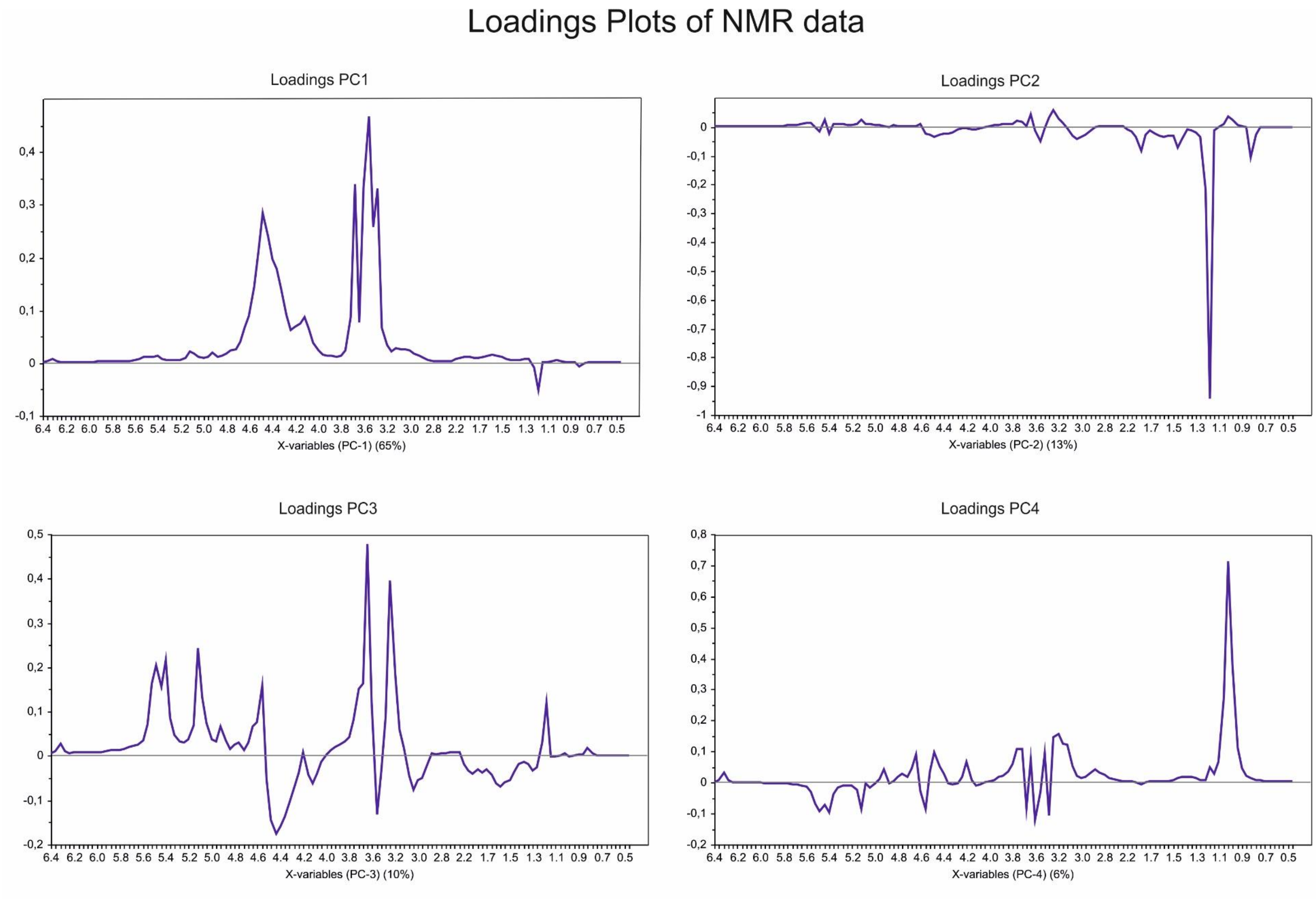

Fig. S5 Loadings Plots of the first four principal components of the PCA of NMR data with the help of which the excipients could be identified, which correlate mainly with the respective principal component
